# *FSCN1* gene polymorphisms: biomarkers for the development and progression of breast cancer

**DOI:** 10.1038/s41598-017-16196-6

**Published:** 2017-11-21

**Authors:** Chao-Qun Wang, Chih-Hsin Tang, Yan Wang, Lulu Jin, Qian Wang, Xiaoni Li, Gui-Nv Hu, Bi-Fei Huang, Yong-Ming Zhao, Chen-Ming Su

**Affiliations:** 1 Department of Pathology, Affiliated Dongyang Hospital of Wenzhou Medical University, Dongyang, Zhejiang, China; 2 Department of Pharmacology, School of Medicine, China Medical University, Taichung, Taiwan; 3 Graduate Institute of Basic Medical Science, China Medical University, Taichung, Taiwan; 4 Department of Biotechnology, College of Health Science, Asia University, Taichung, Taiwan; 5 Department of Medical Oncology, Affiliated Dongyang Hospital of Wenzhou Medical University, Dongyang, Zhejiang, China; 6 Laboratory of Biomedicine, Affiliated Dongyang Hospital of Wenzhou Medical University, Dongyang, Zhejiang, China; 7 Hefei National Laboratory for Physical Sciences at Microscale and School of Life Sciences, University of Science and Technology of China, Anhui, China; 8 Department of Surgical Oncology, Affiliated Dongyang Hospital of Wenzhou Medical University, Dongyang, Zhejiang, China

## Abstract

Breast cancer is a major cause of cancer mortality worldwide. Fascin-1 (FSCN1) is an actin-binding protein found in mammalian cells, including endothelial, neuronal and mesenchymal cells. FSCN1 overexpression has been indicated in breast cancer patients. However, scant information is available regarding the association between *FSCN1* single nucleotide polymorphisms (SNPs) and the risk or prognosis of breast cancer. We report on the association between 6 SNPs of the *FSCN1* gene (rs56156320, rs8772, rs3801004, rs2966447, rs852479 and rs1640233) and breast cancer susceptibility as well as clinical outcomes in 316 patients with breast cancer and in 222 healthy controls. Carriers of the AC or AC + CC allele of the variant rs56156320 were at greater risk of breast cancer compared with wild-type (AA) carriers. Moreover, carriers of at least one G allele in rs3801004 were likely to progress to stage III/IV disease and lymph node metastasis. Individuals with at least one T allele at *FSCN1* SNP rs2966447 were at higher risk of developing pathologic grade G3 disease. Furthermore, individuals bearing the C/C haplotype at SNPs rs56156320 and rs3801004 had nearly twice the risk of breast cancer. Our results indicate that genetic variations in the *FSCN1* gene may serve as an important predictor of early-stage breast cancer.

## Introduction

Breast cancer is associated with high mortality. Over a million women worldwide are diagnosed with breast cancer every year, and over 500,000 succumb to the disease^1^. Risk factors associated with breast cancer in women include her age, family history, reproductive and gynecologic factors, and lifestyle factors including alcohol consumption and lack of physical activity, amongst others^2^. Women who are at high risk of breast cancer may be advised to maintain their mammography screening schedule, undergo genetic testing, or commence chemoprevention.

Current statistical models for estimating breast cancer risk have limited sensitivity and specificity^2^. Researchers have therefore explored genetic variation associated with breast cancer risk, hoping that single nucleotide polymorphism (SNP) genotyping will more accurately stratify breast cancer risk and guide disease management. Emerging reports indicate an association between SNPs in certain genes and susceptibility to breast cancer, as well as clinicopathologic status. Besides the recognized *BRCA1* and *BRCA2* mutations that markedly increase the risk of developing breast cancer^3,4^, a number of additional low- and moderate-risk susceptibility variants have been identified, including caspase-8 (CASP8), an enzyme involved in apoptosis^5^.

Fascin-1 (FSCN1), an actin-binding protein found in mammalian cells including endothelial, neuronal and mesenchymal cells, is low or absent in normal epithelial cells^6,7^. Upregulation of FSCN1 has been indicated in various cancer cell types such as stomach, colon, lung, ovary and breast^8–12^. Overexpression of FSCN1 significantly promotes colon cancer cell migration and metastasis^13^, while knockdown of FSCN1 in cellular models diminishes cell motility and tumor metastasis in prostate cancer^14^ and oral squamous cancer^15^. Several cytokines, including interleukin-6 and oncostatin M, control fascin expression through the activation of the signal transducer and activator of transcription 3 (STAT3) signaling pathway in breast cancer cells^16^. Recent evidence suggests that aberrant STAT3 signaling promotes breast tumor progression by deregulating the expression of downstream target genes that control angiogenesis, including nuclear factor kappaB (NF-κB) and hypoxia-inducible factor 1 (HIF-1), increasing their binding to the fascin gene promoter to induce its expression^17^. Interestingly, a highly significant correlation has been observed between fascin expression and decreased overall survival in African American women with triple-negative breast cancer (TNBC)^18^. Similarly, we have previously described how strong positive FSCN1 expression can be used as a diagnostic marker of TNBC in Chinese women^19^. However, any association between *FSCN1* SNPs with breast cancer risk and prognosis remains to be clarified. We therefore conducted a case-control study to evaluate the role of 6 *FSCN1* SNPs in breast cancer susceptibility and clinicopathological features in a cohort of Chinese Han individuals.

## Results

Sociodemographic characteristics and clinical parameters for all study participants are shown in Table 1. Significant between-group differences were observed for age and tobacco use (both *p* < 0.05), but not for alcohol consumption (*p* > 0.05). Most patients (76.9%) had stage I/II breast cancer; 23.1% had stage III/IV disease (Table 1). In addition, the majority of patients were HER2-positive (Table 1).

**Table 1:** Demographic and clinicopathologic characteristics in healthy controls and patients with breast cancer.

Variable	Controls N = 222 (%)	Patients N = 316 (%)	*p* value
Age (years)	Mean ± S.D.	Mean ± S.D.	
	41.50 ± 14.96	53.09 ± 11.33	*p* < 0.05
Cigarette smoking			
No	217 (97.7)	315 (99.7)	
Yes	5 (2.3)	1 (0.3)	*p* < 0.05
Alcohol consumption			
No	209 (94.1)	297 (94.0)	
Yes	13 (5.9)	19 (6.0)	*p* > 0.05
Clinical stage			
I/II		243 (76.9)	
III/IV		73 (23.1)	
Tumor size			
≤T2		300 (94.9)	
>T2		16 (5.1)	
Lymph node status			
N0 + N1		248 (78.5)	
N2 + N3		68 (21.5)	
Distant metastasis			
M0		306 (96.8)	
M1		10 (3.2)	
Histological grade			
G1 + G2		219 (69.3)	
G3		97 (30.7)	
ER status			
Positive		96 (30.4)	
Negative		220 (69.6)	
PR status			
Positive		146 (46.2)	
Negative		170 (53.8)	
HER2			
Positive		199 (63.0)	
Negative		117 (37.0)	

The Mann-Whitney U-test and Fisher’s exact test were used to compare values between controls and patients with breast cancer. *p < 0.05 was statistically significant. ER, estrogen receptor; PR, progesterone receptor; HER2, human epidermal growth factor receptor 2.

The distribution patterns of *FSCN1* genotypes for all participants are shown in Table 2. In the healthy controls, all genotypic frequencies were in Hardy–Weinberg equilibrium (*p* > 0.05). In both patients and controls, most of those with the rs56156320 SNP, the rs2966447 SNP, or the rs852479 SNP were homozygous for the AA genotype, while most of those with the rs8772 SNP, the rs3801004 SNP, or the rs1640233 SNP were homozygous for CC (Table 2). In analyses adjusted for potential confounders, subjects with the AC or AC + CC genotype of the *FSCN1* rs56156320 polymorphism were almost twice as likely as those with AA homozygotes to develop breast cancer (adjusted odds ratio [AOR], 2.060; 95% CI: 1.020–4.157 or AOR, 1.736; 95% CI: 1.105–2.727; *p* < 0.05). There were no significant differences between cases and controls in regard to the frequency of rs8772, rs3801004, rs2966447, rs852479 and rs1640233 polymorphisms (Table 2).

**Table 2:** Distribution frequency of *FSCN1* genotypes in controls and patients with breast cancer.

Variable	Controls N = 222 (%)	Patients N = 316 (%)	OR (95% CI)	AOR (95% CI)
**rs56156320**				
AA	179 (80.6)	231 (73.1)	1.00 (reference)	1.00 (reference)
AC	14 (6.3)	34 (10.8)	1.882 (0.980–3.613)	2.060 (1.020–4.157)*
CC	29 (13.1)	51 (16.1)	1.363 (0.830–2.237)	1.566 (0.914–2.682)
AC + CC	43 (19.4)	85 (26.9)	1.532 (1.011–2.321)*	1.736 (1.105–2.727)*
**rs8772**				
CC	180 (81.1)	251 (79.4)	1.00 (reference)	1.00 (reference)
CT	37 (16.7)	62 (19.6)	1.202 (0.766–1.884)	1.203 (0.743–1.947)
TT	5 (2.3)	3 (0.9)	0.430 (0.102–1.824)	0.551 (0.117–2.610)
CT + TT	42 (18.9)	65 (20.6)	1.110 (0.720–1.711)	1.132 (0.710–1.803)
**rs3801004**				
CC	208 (93.7)	295 (93.4)	1.00 (reference)	1.00 (reference)
CG	14 (6.3)	20 (6.3)	1.007 (0.497–2.040)	1.321 (0.619–2.820)
GG	0 (0.0)	1 (0.3)	—	—
CG + GG	14 (6.3)	21 (6.6)	1.058 (0.526–2.128)	1.362 (0.642–2.888)
**rs2966447**				
AA	170 (76.6)	235 (74.4)	1.00 (reference)	1.00 (reference)
AT	48 (21.6)	74 (23.4)	1.115 (0.738–1.686)	1.039 (0.665–1.621)
TT	4 (1.8)	7 (2.2)	1.266 (0.365–4.393)	0.962 (0.252–3.676)
AT + TT	52 (23.4)	81 (25.6)	1.127 (0.755–1.682)	1.033 (0.670–1.592)
**rs852479**				
AA	161 (72.5)	210 (66.5)	1.00 (reference)	1.00 (reference)
AC	57 (25.7)	94 (29.7)	1.264 (0.858–1.863)	1.187 (0.783–1.799)
CC	4 (1.8)	12 (3.8)	2.300 (0.728–7.264)	1.608 (0.476–5.436)
AC + CC	61 (27.5)	106 (33.5)	1.332 (0.915–1.940)	1.219 (0.813–1.827)
**rs1640233**				
CC	171 (77.0)	233 (73.7)	1.00 (reference)	1.00 (reference)
CT	43 (19.4)	75 (23.7)	1.280 (0.838–1.955)	1.386 (0.877–2.190)
TT	8 (3.6)	8 (2.6)	0.734 (0.270–1.994)	0.740 (0.251–2.186)
CT + TT	51 (23.0)	83 (26.3)	1.194 (0.800–1.783)	1.284 (0.832–1.980)

The odds ratios (ORs) with their 95% confidence intervals (CIs) were estimated by logistic regression analysis. The adjusted odds ratios (AORs) with their 95% CIs were estimated by multiple logistic regression analysis that controlled for smoking, alcohol consumption, and age. *p < 0.05 was statistically significant.

**Figure 1:**
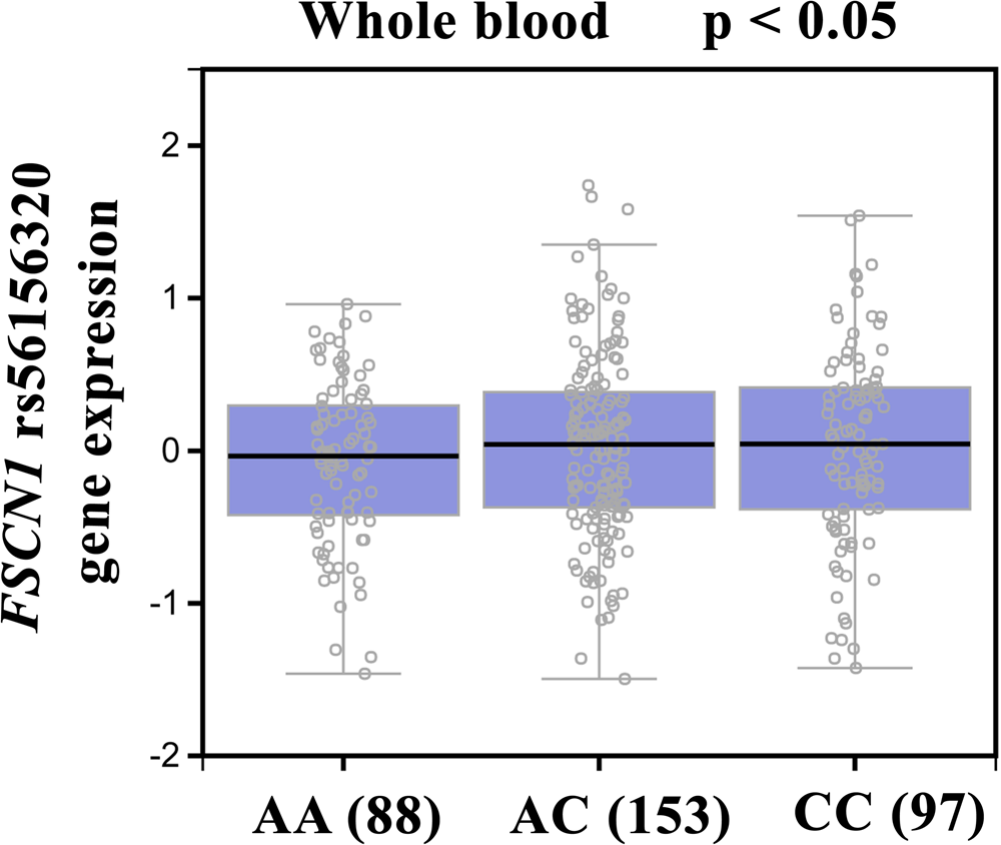
Correlation of rs3219175 genotypes with *FSCN1* mRNA expression in breast cancer whole blood according to the Genotype-Tissue Expression (GTEx) dataset.

We searched the GTEx database to investigate whether rs56156320 was associated with *FSCN1* expression. Those who carried a genotype with the variant C at rs56156320 showed a trend for higher *FSCN1* expression, compared with the wild-type homozygous genotypes (*p* < 0.05, Fig. 1).

Next, we compared the distributions of clinical aspects and *FSCN1* genotypes among cases. Compared with patients with the CC genotype, those with at least one polymorphic allele (CG or GG genotype) at the rs3801004 SNP were at more than twice the risk of developing stage III/IV disease (AOR, 2.540-fold; 95% CI:1.011–6.379) and lymph node metastasis (AOR, 2.804; 95% CI: 1.112–7.070) (Table 3). Moreover, compared with AA carriers, those carrying the AT + TT genotype of rs2966447 were almost twice as likely to develop pathologic grade (G3) disease (AOR, 1.734; 95% CI: 1.016–2.962) (Table 3).

**Table 3:** Odds ratios (ORs) and 95% confidence intervals (CIs) of the clinical status and *FSCN1* rs3801004 and rs2966447 genotypic frequencies in patients with breast cancer.

Gene Genotypes	Patients N = 316 (%)		OR (95% CI)	AOR (95% CI)
Clinical Stage				
**Stage I/II**		**Stage III/IV**		
**rs3801004**				
CC	231 (78.3)	64 (21.7)	1.00 (reference)	1.00 (reference)
CG + GG	12 (57.1)	9 (42.9)	2.707 (1.092–6.709)*	2.540 (1.011–6.379)*
**rs2966447**				
AA	183 (77.9)	52 (22.1)	1.00 (reference)	1.00 (reference)
AT + TT	60 (74.1)	21 (25.9)	1.232 (0.686–2.210)	1.275 (0.707–2.300)
**Tumor size**				
	**≦T2**	**>T2**		
**rs3801004**				
CC	281 (95.3)	14 (4.7)	1.00 (reference)	1.00 (reference)
CG + GG	19 (90.5)	2 (9.5)	2.113 (0.447–9.982)	1.958 (0.402–9.531)
**rs2966447**				
AA	222 (94.5)	13 (5.5)	1.00 (reference)	1.00 (reference)
AT + TT	78 (96.3)	3 (3.7)	0.657 (0.182–2.366)	0.693 (0.191–2.507)
**Lymph node metastasis**				
	**N0 + N1**	**N2 + N3**		
**rs3801004**				
CC	236 (80.0)	59 (20.0)	1.00 (reference)	1.00 (reference)
CG + GG	12 (57.1)	9 (42.9)	3.000 (1.207–7.454)*	2.804 (1.112–7.070)*
**rs2966447**				
AA	187 (79.6)	48 (20.4)	1.00 (reference)	1.00 (reference)
AT + TT	61 (75.3)	21 (24.7)	1.277 (0.704–2.319)	1.324 (0.725–2.419)
**Distant metastasis**				
	**M0**	**M1**		
**rs3801004**				
CC	286 (93.5)	9 (3.1)	1.00 (reference)	1.00 (reference)
CG + GG	20 (95.2)	1 (4.8)	1.589 (0.192–13.173)	2.395 (0.275–20.879)
**rs2966447**				
AA	226 (96.2)	9 (3.8)	1.00 (reference)	1.00 (reference)
AT + TT	80 (98.8)	1 (1.2)	0.314 (0.039–2.517)	0.295 (0.037–2.379)
**Pathologic grade**				
	**G1 + G2**	**G3**		
**rs3801004**				
CC	203 (68.8)	92 (31.2)	1.00 (reference)	1.00 (reference)
CG + GG	16 (76.2)	5 (23.8)	0.690 (0.245–1.939)	0.794 (0.278–2.268)
**rs2966447**				
AA	171 (72.8)	64 (27.2)	1.00 (reference)	1.00 (reference)
AT + TT	48 (59.3)	33 (40.7)	1.837 (1.083–3.115)*	1.734 (1.016–2.962)*

The ORs with their 95% CIs were estimated by logistic regression analysis. The adjusted odds ratios (AORs) with their 95% confidence intervals (CIs) were estimated by multiple logistic regression analysis that controlled for smoking, alcohol consumption, and age. *p < 0.05 was statistically significant.

We further analyzed the clinical aspects and rs2966447 *FSCN1* genotypic frequencies in different breast cancer subtypes. Individuals in the Luminal A subgroup who had at least one G allele were at higher risk of developing stage III/IV disease and lymph node metastasis (Table 4). Similarly, those in the Luminal B subgroup with at least one G allele were more likely to develop pathologic grade (G3) disease.

**Table 4:** Odds ratios (ORs) and 95% confidence intervals (CIs) of the clinical status and *FSCN1* rs2966447 genotypic frequencies in patients with breast cancer.

**Variable**	**Luminal A**			**Luminal B**			**HER2 overexpression**			**TNBC**		
	**AA N=76(%)**	**AT+TT N=28(%)**	**OR (95% CI)**	**AA N=84(%)**	**AT+TT N=33(%)**	**OR (95% CI)**	**AA N=46(%)**	**AT+TT N=12(%)**	**OR (95% CI)**	**AA N=34(%)**	**AT+TT N=8(%)**	**OR (95% CI)**
**Clinical stage**												
**Stage I/II**	67 (88.2)	20 (71.4)	1.00 (reference)	60 (71.4)	26 (78.8)	1.00 (reference)	30 (65.2)	8 (66.7)	1.00 (reference)	26 (89.7)	6 (75.0)	1.00 (reference)
**Stage III/IV**	9 (11.8)	8 (28.6)	2.978 (1.016–8.728)*	24 (28.6)	7 (21.2)	0.673 (0.258**–**1.757)	16 (34.8)	4 (33.3)	0.938 (0.244**–**3.598)	3 (10.3)	2 (25.0)	2.889 (0.392**–**21.289)
**Tumor size**												
**≦ T2**	76 (100)	27 (96.4)	1.00 (reference)	78 (92.9)	33 (100)	1.00 (reference)	40 (87.0)	11 (91.7)	1.00 (reference)	28 (96.6)	7 (87.5)	1.00 (reference)
**>T2**	0 (0)	1 (3.6)	—	6 (7.1)	0 (0)	—	6 (13.0)	1 (8.3)	0.606 (0.066**–**5.578)	1 (3.4)	1 (12.5)	4.000 (0.222**–**72.183)
**Lymph node status**												
**N0+N1**	67 (88.2)	20 (71.4)	1.00 (reference)	60 (71.4)	26 (78.8)	1.00 (reference)	34 (73.9)	9 (75.0)	1.00 (reference)	26 (89.7)	6 (75.0)	1.00 (reference)
**N2+N3**	9 (11.8)	8 (28.6)	2.978 (1.016–8.728)*	24 (28.6)	7 (21.2)	0.673 (0.258**–**1.757)	12 (26.1)	3 (25.0)	0.944 (0.219**–**4.079)	3 (10.3)	2 (25.0)	2.889 (0.392**–**21.289)
**Distant metastasis**												
**M0**	75 (98.7)	27 (96.4)	1.00 (reference)	82 (97.6)	33 (100)	1.00 (reference)	42 (91.3)	12 (100)	1.00 (reference)	27 (93.1)	8 (100)	1.00 (reference)
**M1**	1 (1.3)	1 (3.6)	2.778 (0.168**–**45.977)	2 (2.4)	0 (0)	—	4 (8.7)	0 (0)	—	2 (6.9)	0 (0)	—
**Pathologic grade**												
**G1+G2**	74 (97.4)	25 (89.3)	1.00 (reference)	64 (71.4)	18 (54.5)	1.00 (reference)	23 (50.0)	2 (16.7)	1.00 (reference)	10 (34.5)	3 (37.5)	1.00 (reference)
**G3**	2 (2.6)	3 (10.7)	4.440 (0.701**–**28.118)	24 (28.6)	15 (45.5)	2.667 (1.140–6.236)*	23 (50.0)	10 (83.3)	5.000 (0.985**–**25.379)	19 (65.5)	5 (62.5)	0.877 (0.173**–**4.447)

The ORs with their 95% CIs were estimated by logistic regression analysis. *p < 0.05 was statistically significant. HER2, human epidermal growth factor receptor 2; TNBC, triple-negative breast cancer. Pathological grade: G1, well differentiated; G2, moderately differentiated; G3, poorly differentiated.

**Figure 2:**
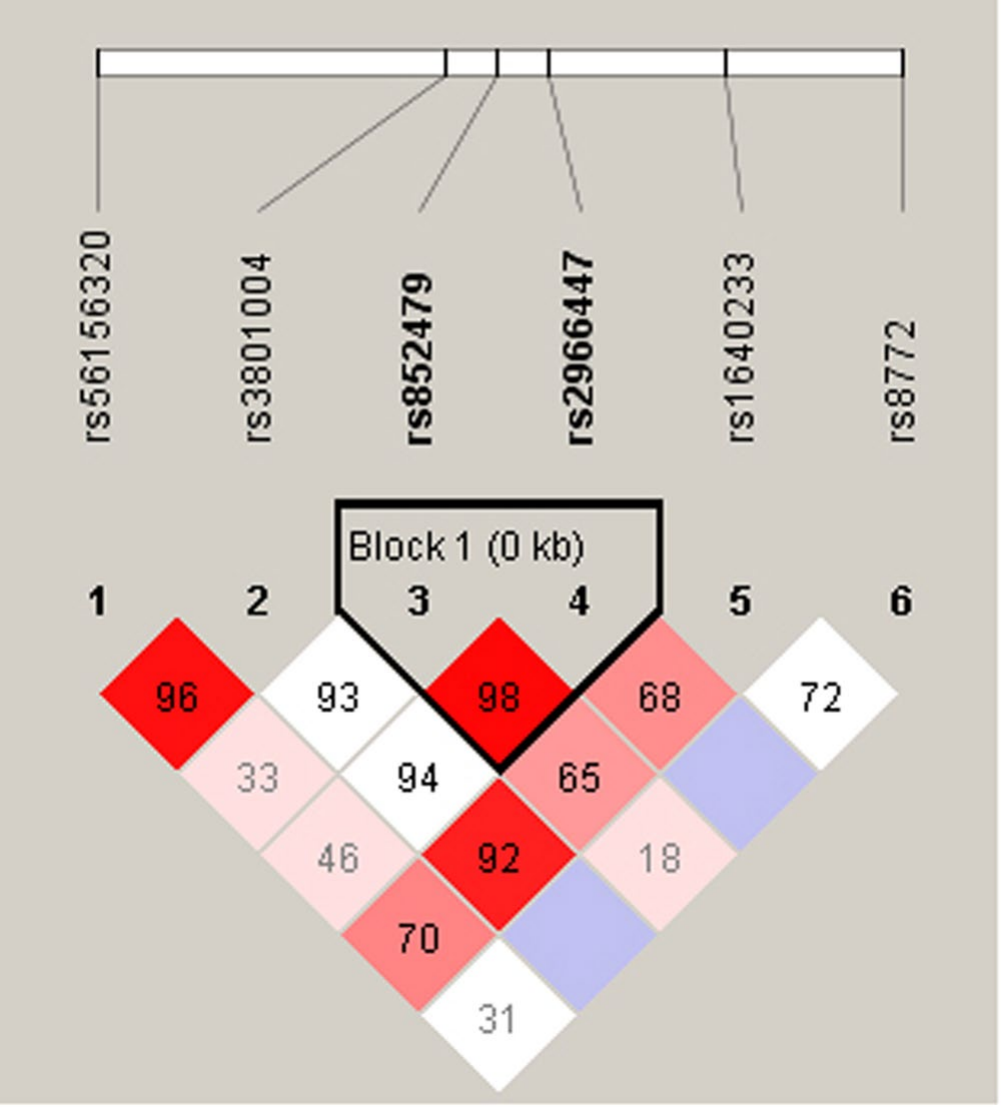
Linkage disequilibrium (LD) map for single nucleotide polymorphisms in the *FSCN1* gene. There are 538 participants, including 222 healthy people and 316 patients with breast cancer, in this study. Block is pairwise D’ plots and haplotype blocks obtained from HAPLOVIEW.

An analysis of *FSCN1* rs56156320 and rs3801004 haplotype distribution frequencies revealed that the most common haplotype in healthy controls was AC (83.8%), which was therefore selected as the reference. The C-C *FSCN1* haplotype significantly increased the risk for developing breast cancer by almost 2-fold compared with the reference group A-C (*p* < 0.05) (Table 5). The reconstructed linkage disequilibrium plot of the 4 SNPs is shown in Fig. 2. We found a haploblock in which rs56156320 and rs3801004 showed 96% linkage disequilibrium. In addition, rs852479 and rs2966447 as well as rs3801004 and rs1640233 expressed 98% and 92% linkage disequilibrium, respectively (Fig. 2).

**Table 5:** Distribution frequency of *FSCN1* haplotypes in healthy controls and patients with breast cancer.

Haplotype block		Controls N = 444 (%)	Patients N = 632 (%)	OR (95% CI)	AOR (95% CI)
rs56156320	rs3801004				
A/C	C/G				
**A**	C	372 (83.8)	495 (78.3)	Reference	
C	C	58 (13.1)	115 (18.2)	1.490 (1.057–2.100)*	1.674 (1.150–2.437)*
C	G	14 (3.2)	21 (3.3)	1.127 (0.566–2.246)	1.386 (0.662–2.901)
A	G	0 (0)	1 (0.2)	**─**	─

The odds ratios (ORs) with their 95% confidence intervals (CIs) were estimated by logistic regression analysis. The adjusted odds ratios (AORs) with their 95% CIs were estimated by multiple logistic regression analysis that controlled for smoking, alcohol consumption, and age. *p < 0.05 was statistically significant.

## Discussion

FSCN1, a 55-kDa cytoskeletal actin-binding protein that packages actin filaments into tertiary structures, including microspikes, stress fibers and membrane ruffles, within dynamic cellular structures, enhances cell motility, migration and adhesion^20^. FSCN1 is widely expressed in the developing nervous system, whereas in normal adult tissue, FSCN1 is highly restricted to antigen-presenting dendritic cells, endothelial cells, glial cells and neurons^21^. It has been suggested that fascin is overexpressed or upregulated in various human cancers, such as colon, lung, stomach and breast^22–24^. In addition, fascin expression plays a central role in regulating breast cancer cell morphology, migration and invasion potential^24^. Inhibition of FSCN1 reduces cancer migration and tumor metastasis in prostate and oral squamous cancer cells^14,15^. Furthermore, docosahexaenoic acid reduces FSCN1-dependent breast cancer metastasis^25^. These results suggest that knockdown FSCN1 might be a valuable therapeutic strategy for breast cancer.

Breast cancer is the most commonly diagnosed neoplasm and the third leading cause of cancer-associated mortality in the United States, with 22.2 mortalities per 100,000 women associated with breast cancer each year. The 5-year relative survival rate for breast cancer has gradually increased since the early 1990s; between 2007 and 2011 it was ~89.2%. The prognosis of patients with breast cancer is critically dependent on the disease stage at the time of diagnosis. Therefore, it is important to increase screening rates and genetic testing for hereditary breast cancer, to increase the chances of early diagnosis^26,27^. To the best of our knowledge, this study is the first to examine the distribution of the rs56156320, rs8772, rs3801004, rs2966447, rs852479 and rs1640233 SNPs and their possible association with breast cancer development. We also investigated the associations of these *FSCN1* SNPs with clinical status, clinical pathologic markers, and susceptibility for breast cancer. In analyses adjusted for potential confounding factors, patients who had AC or AC + CC genotype in the rs56156320 SNP were at increased risk of developing breast cancer, by 2.060- or 1.736-fold (95% CI: 1.020–4.157 or 95% CI: 1.105–2.727; *p* < 0.05). No significant differences were observed between patients and healthy controls in the frequencies of the rs8772, rs3801004, rs2966447, rs852479 and rs1640233 polymorphisms. The polymorphisms in the 3ʹ-flanking region of a gene can control gene expression^28^. Data from the GTEx database demonstrated that variant C at rs56156320 showed a trend for increased expression of *FSCN1*, compared with the wild-type AA homozygous genotypes. This result confirms our SNP data and indicates that the *FSCN1* rs56156320 SNP may control the expression of FSCN1.

This study found that breast cancer patients with the *FSCN1* rs3801004 polymorphism had a higher risk of developing stage III/IV disease and lymph node metastasis. Similarly, the *FSCN1* rs2966447 polymorphism was also associated with a higher risk of developing pathological grade (G3) disease. Interestingly, the rs2966447 SNP was associated with a higher risk of developing stage III/IV disease and lymph node metastasis in the Luminal A subgroup, as well as a higher risk of pathologic grade (G3) disease in the Luminal B subgroup. It is established that overexpression of the *FSCN1* gene is implicated in the development and metastasis of breast cancer^29^. In addition, FSCN1 is involved in the chemotherapeutic resistance of breast cancer cells^30^. However, more research is required to determine whether an association exists among advanced-stage disease, FSCN1 expression levels and *FSCN1* genotype, and clarification is needed in regard to the effects of the *FSCN1* genotype on breast cancer risk.

Linkage disequilibrium is expressed across the human genome. Thus, loci can be used as genetic markers to locate adjacent variants that participate in the detection and treatment of disease. Haplotype analyses can provide data on the genetic contribution to disease susceptibility^31^. We evaluated the impacts of different haplotype combinations of 2 *FSCN1* SNPs (rs56156320 and rs3801004) upon the risk of breast cancer and found that the CC haplotype was associated with a higher risk for breast cancer. It is possible that the *FSCN1* CC haplotype is in linkage disequilibrium with other functional polymorphisms that are responsible for susceptibility to breast cancer. On the other hand, we also found that rs56156320 and rs3801004 showed 96% linkage disequilibrium. Furthermore, rs852479 and rs2966447 as well as rs3801004 and rs1640233 expressed 98% and 92% linkage disequilibrium, respectively. These results suggest that these *FSCN1* haplotypes play an important role in breast cancer development.

In conclusion, our results demonstrate an association between *FSCN1* gene variants and the risk of breast cancer. We show that the *FSCN1* rs56156320 polymorphisms significantly increase the risk of breast cancer progression among Chinese Han females. This study is the first to report a correlation between *FSCN1* polymorphisms and breast cancer risk. *FSCN1* may serve as a predictive marker for breast cancer therapy.

## Materials And Methods

### Participants

Between 2014 and 2016, we collected 318 blood specimens from patients (cases) who had been diagnosed with breast cancer at Dongyang People’s Hospital. The control group consisted of 222 healthy participants without a history of cancer. All participants provided written informed consent, and the study was approved by the Ethics Committee of Dongyang People’s Hospital. This study’s protocol was approved by the Ethics Committee of Dongyang People’s Hospital and all experiments were performed in accordance with relevant guidelines and regulations. Pathohistologic diagnosis followed the World Health Organization classification of breast tumors and tumors were graded using the Scarff-Bloom-Richardson method^32^. Breast cancer cases were categorized by estrogen receptor (ER), progesterone receptor (PR), human epidermal growth factor receptor 2 (HER2) and Ki‐67 status, into 4 subtypes: Luminal A (ER^+^ and/or PR^+^, HER2^**−**^, Ki‐67 < 14%); Luminal B (ER^+^ and/or PR^+^ , HER2^**−**^, Ki‐67 ≥ 14%; or ER^+^ and/or PR^+^, HER2^+^); HER2‐enriched (ER^**−**^, PR^**−**^, HER2^+^); or TNBC (ER^**−**^, PR^**−**^, HER2^**−**^). A standardized questionnaire and the electronic medical record system were searched for demographic data on age, sex, smoking history, and alcohol consumption.

### Determination of genotypes

Total genomic DNA was isolated from whole blood specimens using QIAamp DNA blood mini kits (Qiagen, Valencia, CA), as per the manufacturer’s instructions. The DNA was dissolved in Tris-EDTA (TE) buffer composed of 10 mM Tris-HCl containing 1 mM EDTA•Na_2_ (pH 7.8) and stored at −20 °C until it was subjected to quantitative polymerase chain reaction (PCR) analysis. Six *FSCN1* SNPs (rs56156320, rs8772, rs3801004, rs2966447, rs852479 and rs1640233) were examined with the use of a commercially available TaqMan SNP genotyping assay (Applied Biosystems, Warrington, UK), according to the manufacturer’s protocols^33,34^.

### Bioinformatic analysis

Genotype-Tissue Expression (GTEx) data were used to identify correlations between SNPs and levels of FSCN1 expression^35^. We conducted an investigation of expression quantitative trait loci (eQTLs), to determine the functional role of phenotype-associated SNPs.

### Statistical analysis

The genotype distribution of each SNP was analyzed for Hardy–Weinberg equilibrium and confirmed by Chi-square analysis. Demographic characteristics were compared between patients and controls using the Mann–Whitney U-test and Fisher’s exact test. Associations between genotypes, breast cancer risk and clinicopathologic characteristics were estimated using adjusted odds ratios (AORs) and 95% confidence intervals (CIs), after controlling for other covariates. Significant differences in haplotype frequencies between cases and controls were analyzed using Haploview, according to the software package^36^. A *p* value of <0.05 was considered statistically significant. Data were analyzed using SAS statistical software (Version 9.1, 2005; SAS Institute Inc., Cary, NC).
